# Spontaneous traumatic macular hole closure in a 50-year-old woman: a case report

**DOI:** 10.1186/1752-1947-5-290

**Published:** 2011-07-06

**Authors:** Mayssa B Nasr, Chrysanthos Symeonidis, Ioannis Tsinopoulos, Sofia Androudi, Tryfon Rotsos, Stavros A Dimitrakos

**Affiliations:** 1 2nd Department of Ophthalmology, "Papageorgiou" General Hospital, School of Medicine, Aristotle University of Thessaloniki, Greece; 2 Department of Ophthalmology, Leicester Royal Infirmary, University Hospitals of Leicester NHS Trust, UK; 3 Department of Ophthalmology, School of Medicine, University of Thessaly, Greece

## Abstract

**Introduction:**

Traumatic macular holes (TMH) are well-known complications of ocular contusion injury. Spontaneous closure occurs in approximately 50% of cases, but rarely after the age of thirty. We report a case of spontaneous closure of a full thickness macular hole due to a blunt trauma and we suggest possible mechanisms for this closure.

**Case presentation:**

A 50-year-old Greek woman was referred with a history of reduced best-corrected visual acuity after blunt trauma to her right eye. Diagnosis was based on fundoscopic, optical coherence tomography as well as fluorescein angiography findings with follow-up visits at two days, 20 days and five months. Fundoscopy revealed a full-thickness TMH with a minor sub-retinal hemorrhage and posterior vitreous detachment. The presence of a coagulum in the TMH base was observed. Subsequently, TMH closure was observed.

**Conclusion:**

The clot in the TMH base, potentially a hemorrhage by-product containing a significant quantity of platelets, may have simulated the clot observed after autologous serum use, thus facilitating a similar effect. This may have stimulated glial cell migration and proliferation, thus contributing to spontaneous hole closure.

## Introduction

Commotio retinae, diffuse retinal edema, retinal hemorrhage, vitreous hemorrhage, choroidal rupture, photoreceptor injury, and macular holes (MH) are well known complications of ocular contusion injury. According to relevant literature, the frequency of traumatic macular holes (TMH) is between 1% and 9% [1]. The major cause of blunt trauma is sports-related accidents such as baseball and soccer ball, thus the higher frequency of TMH in younger patients. TMH is thought to occur as an immediate concussive tear or as a belated breakdown of traumatically induced cystoid change. Immediate visual loss after injury is probably due to retinal dehiscence on concussion, whereas delayed visual loss is likely to indicate a secondary event of vitreoretinal interface changes. Vitrectomy and fluid-gas exchange is a current management for the repair of TMH. Spontaneous closure occurs in approximately 50% of cases, but rarely after the age of thirty [2].

## Case Presentation

**Figure 1:**
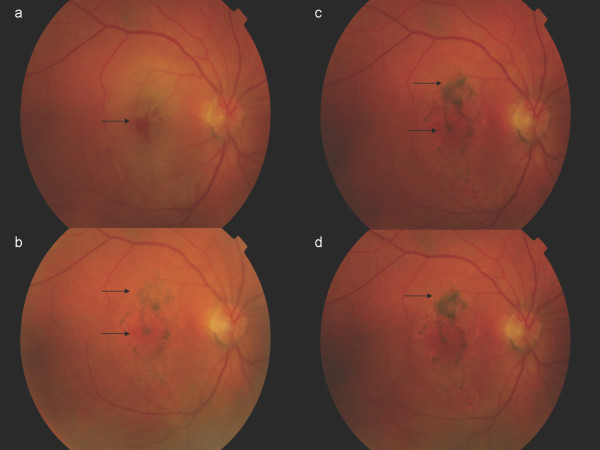
**Colour fundus**. Three days after the blunt trauma. At 20th day follow-up visit. At three-month follow-up visit; at four-month follow-up visit.

**Figure 2:**
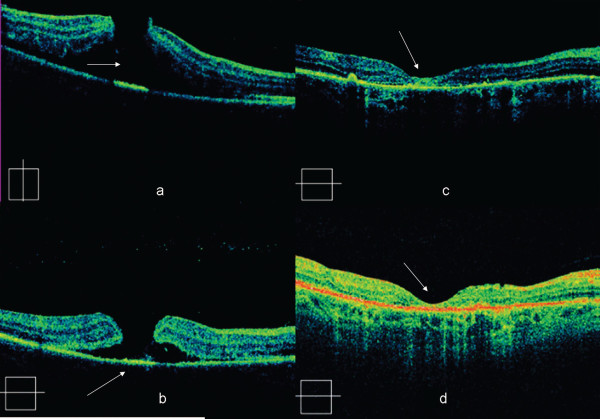
**Cirrus OCT scans**. One hour after injury depicting a full thickness MH with surrounding neurosensory retinal detachment. At the third day follow-up visit, depicting a coagulum covering the TMH base. At the 20th day follow-up visit depicting a resolution of the MH with remaining surrounding neurosensory retinal detachment. Retinal thickness was 216 μm at the level of the fovea, At the four-month follow-up, with a complete resolution of the MH and surrounding neurosensory retinal detachment. Retinal thickness was 235 μm at the level of the fovea.

A 50-year-old Greek woman was referred to us with a history of reduced best-corrected visual acuity (BCVA) after blunt trauma to her right eye. Past medical and ocular history was unremarkable. Her BCVA one hour after the incident was hand movements at 10 cm. An anterior chamber examination revealed a round pupil with no signs of hyphema or iridodialysis. Fundoscopy revealed a full-thickness TMH with a minor sub-retinal hemorrhage and posterior vitreous detachment (PVD, Figure 1). Cirrus optical coherence tomography (OCT) scans confirmed the funduscopic findings (Figure 2).

**Figure 3:**
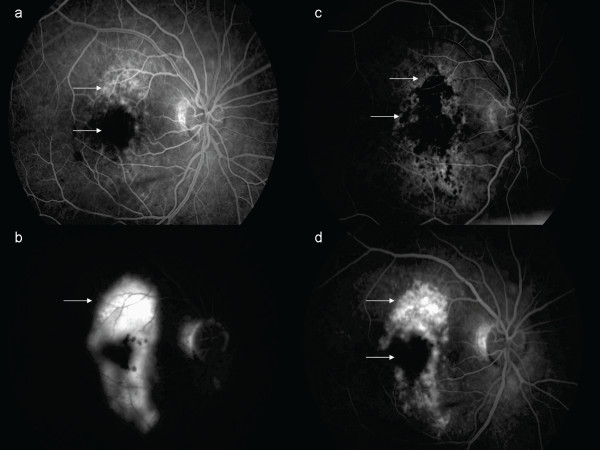
**Fluorescein angiography**. Three days after blunt force trauma. Arteriovenous phase; window defect peripheral to the macula, in addition to central masking due to sub-retinal hemorrhage. Three days after blunt trauma. Late phase angiogram; perimacular pooling of the injected dye. At the three-month follow-up visit. Arterious phase; window defect and masking due to hyper- and hypopigmentation. Three-month follow-up visit. Late phase; perimacular staining, resolution of the MH.

Two days later, her BCVA was improved (counting fingers at 1.5 m), despite a foveal hemorrhage. Fluorescein angiography (FA) revealed masking in the fovea, progressive staining peripheral to the masking area and a central window defect (Figure 3). OCT examination showed a marked decrease in retinal edema and a coagulum covering the TMH base (Figure 2).

Seventeen days later, her BCVA had improved to 7 Early Treatment Diabetic Retinoapthy Study (ETDRS) letters at 4 m. Fundoscopy and OCT scans revealed TMH closure and adjacent pigment dispersion (Figures 1, 2). Four months later, with a BCVA of 15 ETDRS letters at 4 m, our patient was fixating eccentrically, despite BCVA improvement. Fundoscopy showed hyper- and hypo-pigmentation in the TMH periphery and an OCT scan confirmed the TMH closure (Figures 1, 2). FA revealed an absence of central window defect and mottled hyper-fluorescence consistent with diffuse retinal pigment epithelial (RPE) atrophy (Figure 3).

## Discussion

TMH is a rare complication of blunt trauma; contrary to idiopathic MHs, it usually has a lamellar configuration [3]. Spontaneous TMH closure is common, but infrequent in patients over thirty.

Yamashita *et al*. [4] proposed two distinct mechanisms of TMH formation, depending on whether the posterior hyaloid is attached or detached. One type causes immediate visual loss due to primary dehiscence of the fovea. The other type leads to delayed visual loss due to dehiscence of the fovea secondary to persistent vitreofoveal adhesion. In older patients, posterior vitreous is usually detached, making TMH in general less frequent in older patients.

Our patient shared common features with similar previously reported cases; an observed full-thickness TMH, and a limited BCVA improvement [2], despite hole closure. The latter may be explained by contusion damage which causes irreversible photoreceptor and RPE damage. The distinct feature of this patient is the relatively old age (for spontaneous closure), and the possible mechanism for TMH resolution. Several authors have suggested autologous serum as an adjuvant to vitrectomy for MH surgical management. The serum beneficial effect may be due to the presence of growth factors (GF), such as GF-platelet-derived GF, epidermal GF, and insulin-like GF-1, and cytokines, which have been shown to promote wound healing [5]. Additionally, autologous serum has been shown to be chemotactic for glial and RPE cells. Following autologous serum injection in idiopathic MHs, a white coagulum may cover the hole, in many cases for one to two weeks. Even if this may be merely platelet clumping, it is conceivable that it contains clotting factors (for example fibrin) that may have a beneficial effect by providing a scaffold for cell proliferation, thus promoting hole closure by mechanical means [5].

## Conclusion

In our case, the clot in the TMH base, potentially a hemorrhage by-product containing a significant quantity of platelets, may have simulated the clot observed after autologous serum use, thus facilitating a similar effect. This may have stimulated glial cell migration and proliferation, thus contributing to spontaneous hole closure.

## Consent

Written informed consent was obtained from the patient for publication of this case report and any accompanying images. A copy of the written consent is available for review by the Editor-in-Chief of this journal.

## Competing interests

The authors declare that they have no competing interests.

## Authors' contributions

MBN was involved in data acquisition and manuscript drafting. CS was involved in manuscript drafting. IT was involved in data acquisition and manuscript drafting. SA revised the report critically for important intellectual content. TR was involved in data interpretation and revised the report critically for important intellectual content. SAD gave final approval of the version to be published. All authors read and approved the final manuscript.

## References

[B1] Querques G Barone A Forte R Prascina F Iaculli C Delle Noci N Optical coherence tomography and fundus-related perimetry in spontaneous closure of a traumatic macular hole J Fr Ophtalmol 2008 31 710 713 10.1016/S0181-5512(08)74386-9 18971857

[B2] Bosch-Valero J Mateo J Lavilla-García L Núñez-Benito E Cristóbal JA Spontaneous closure of full thickness traumatic macular holes Arch Soc Esp Oftalmol 2008 83 325 327 18464183 10.4321/s0365-66912008000500009

[B3] Gill MK Lou PL Traumatic macular holes Int Ophthalmol Clin 2002 42 97 106 10.1097/00004397-200207000-00012 12131587

[B4] Yamashita T Uemara A Uchino E Doi N Ohba N Spontaneous closure of traumatic macular hole Am J Ophthalmol 2002 133 230 235 10.1016/S0002-9394(01)01303-4 11812427

[B5] Minihan M Goggin M Cleary PE Surgical management of macular holes: results using gas tamponade alone, or in combination with autologous platelet concentrate, or transforming growth factor beta 2 Br J Ophthalmol 1997 81 1073 1079 10.1136/bjo.81.12.1073 9497468 PMC1722094

